# Quantum correlation of qubit-reservoir system in dissipative environments

**DOI:** 10.1038/s41598-017-07235-3

**Published:** 2017-08-17

**Authors:** Tao Wu, Jiadong Shi, Lizhi Yu, Juan He, Liu Ye

**Affiliations:** 1 School of Physics & Electronics Engineering, Fuyang Normal University, Fuyang, 236037 China; 2 Research Centre of Quantum Information Technology, Fuyang Normal University, Fuyang, 236037 China; 3 School of Physics & Material Science, Anhui University, Hefei. Anhui, 230601 China

## Abstract

In this work, the dynamics of quantum correlation (QC) in terms of geometric discord and its transfer coupled with dissipative reservoirs are investigated. Taken two canonical cases where the qubits of interest are initially prepared in extended Werner-like state and W-like states into account, we specifically reveal the dynamical behaviors of the geometric discord as each qubit locally interacts with its surrounding infinite degree-of-freedom reservoir. In the scenarios, the short-term and long-term dynamics of the geometric discord for the qubit- and reservoir-subsystem as well as its transfers between them are observed detailedly. It turns out that the geometric discord of qubit-subsystem decays asymptotically to zero while the counterpart of reservoir-subsystem can revive from time *t* = 0 to steady value, which sheds light on a transfer of the discord from the qubit-subsystem to the corresponding reservoir-subsystem.

## Introduction

Over the last decade, entanglement plays an important role in the region of quantum information theory^1^. However, it has been found that entanglement is not the only kind of nonclassical correlation that can exist between systems, i.e., quantum systems in mixed states can be disentangled and yet still nonclassically correlated^2–5^. These correlations can be quantified by quantum discord, which has been demonstrated theoretically^6^ and experimentally^7^ that some separable states can improve performance in some computational tasks over their classical counterparts. Quantum discord, as a measure of nonclassically correlations with respect to a bipartite quantum state, which was initially introduced by Ollivier and Zurek^4^ and by Henderson and Vedral^7^, has attracted considerable attention^8–26^.

Now, let us briefly review the definition of quantum discord for a bipartite quantum state ${\rho }^{AB}$ on ${H}^{A}\otimes {H}^{B}$ with marginals ${\rho }^{A}$ and ${\rho }^{B}$, which can be expressed as ref. 11

1 $$Q(\rho )\,:=\mathop{{\rm{\min }}}\limits_{{{\rm{\Pi }}}^{A}}\{I(\rho )-I[{{\rm{\Pi }}}^{A}(\rho )]\},$$

where the minimum is taken over von Neumann measurements (one-dimensional orthogonal projectors summing to the identity) ${{\rm{\Pi }}}^{A}=\{{{\rm{\Pi }}}_{j}^{A}\}$ on party *A*, and

2 $${{\rm{\Pi }}}^{A}(\rho )\,:=\sum _{j}({{\rm{\Pi }}}_{j}^{A}\otimes {I}^{B})\rho ({{\rm{\Pi }}}_{j}^{A}\otimes {I}^{B})$$

is the post-measurement state, *I* ^*B*^ is the identity operator on the Hilbert space of qubit *B*, and

3 $$I({\rho }^{XY})=S({\rho }^{X})+S({\rho }^{Y})-S({\rho }^{XY})$$

is denoted as the mutual information with the von Neumann entropy $S(\rho )=-tr(\rho {\mathrm{log}}_{2}\rho )$. The intuitive meaning of quantum discord thus may be interpreted as the minimal loss of correlations (as measured by the quantum mutual information) induced by the measurement. However, the quantum discord needs minimization procedure for quantum measurements, and its analytical result is only known for a few classes of two-qubit states. Accordingly, some different methods of computing the discord in qubit system have been put forward, such as the geometric measure of quantum discord^11, 27, 28^, and so on. The geometric discord is defined as the trace distance correlation between the system state and its closest classical state^16^. The geometric discord involves an analytical formula and more computable for the general two-qubit system in contrast to quantum discord and many efforts have been made for unveiling its dynamic behaviors.

The realistic quantum systems suffer from inevitable interactions with their surrounding environments. These undesired interactions will lead to decoherence and dissipation that exponentially damages the quantum correlation (QC). Therefore, the study of the dynamics of quantum discord for an open quantum system has attracted extensive studies, in theoretical^29–38^ and experimental^39, 40^ aspects. For instance, the quantum discord can accurately detect the phase transitions of the condensed matter systems which had been verified by the refs 35–38. In this paper, we investigate the dynamics of QC in terms of geometric discord and its transfer in dissipative reservoirs. We consider two different models: two qubits *A* and *B* are initially prepared in an extended Werner-like state^41^ and three qubits *A*, *B* and *C* are initially in an extended *W*-class state^42^ each locally interacting with its own multi-mode reservoirs, respectively. The extended Werner-like states are the mixed states composed of the totally mixed state, Bell-like pure state and separable state under the different conditions, so one can probe the dynamics of quantum correlation using the different entanglement. In both models, we develop the short-term and long-term dynamics of geometric discord and its transfer from the qubit-subsystem to the corresponding reservoir-subsystem. It shows that the geometric discord of qubit-subsystem decays asymptotically to zero and that of reservoir-subsystem revives from time *t* = 0, which displays a clear transfer of the discord from the qubit- to the reservoir-subsystem. Intriguingly, the discords in both of the cases undergo sudden changes during the evolution except that for the case of $\alpha =\beta =1/2,\gamma =\sqrt{2}/2$ (*α*, *β* and *γ* are state parameters of W-class state) in the second model. Finally, the long-term behaviors of geometric discord for the qubit-subsystem decays asymptotically to zero and that for the reservoir-subsystem revives from zero to steady value. In comparison with the sudden death and sudden birth of qubits’ and reservoirs’ entanglements which behaviors have been demonstrated in ref. 43, the corresponding discord always evolves asymptotically. In this sense, the discord is more robust against dissipative environments compared with the entanglement, and this robustness is in essence because of the ingredient of quantum correlation other than entanglement. In addition, the entanglement doesn’t subject to the sudden change during the evolution. Here, it should be emphasized that the similar dynamic behaviors of QC have been demonstrated by means of the geometric discord in contrast to that of the entropic discord.

## Results

### The dynamics of geometric discord and its transfer for Werner state

Let us firstly focus on the dynamics of geometric discord for the Werner state coupled with a Lorentzian structured reservoir that is dependent on the chosen effective spectral density of the intracavity field. The descriptions of geometric discord and of the interactional Lorentzian structured reservoir will be presented in the Methods section. Herein, we consider a bipartite qubit-system consisting of two non-interacting atoms *A* and *B* each locally interacting its own multimode reservoirs labeled as *a* and *b*. Assume that the reservoirs *a* and *b* are initially in the vacuum state ${|\bar{0}\bar{0}\rangle }_{ab}$, and the two atoms are initially prepared in an extended Werner-like (EWL) state^41^ defined by

4 $${\rho }_{AB}(0)=\frac{1-p}{4}I+p|\varphi \rangle \langle \varphi |,$$

where *I* is a 4 × 4 identity matrix, the complex numbers *α* and *β* are the state parameters with $|\varphi \rangle =\alpha |00\rangle +\beta |11\rangle$, and satisfy the normalized condition ${|\alpha |}^{2}+{|\beta |}^{2}=1$, and the parameter *p* is real-valued which indicates the purity of initial state, i.e. *p* is 1 for pure sate and 0 for completely mixed state.

For simplicity, we will derive the evolution of the overall qubit-reservoir system and the corresponding explicit form of ${\rho }_{ABab}(t)$ as it can be easily obtained via the methods previously mentioned^43, 44^ in the Supplementary Information. To calculate the geometric discord of the qubit-subsystem *AB*, we could render the five characterizing parameters of the reduced density matrix ${\rho }_{AB}(t)$ in the Bloch decomposition

5 $$x=p\{{\alpha }^{2}+{\beta }^{2}[{|{\xi }_{1}(t)|}^{4}-{|{\xi }_{0}(t)|}^{4}]\},$$

6 $$y=p\{{\alpha }^{2}+{\beta }^{2}[{|{\xi }_{1}(t)|}^{4}-{|{\xi }_{0}(t)|}^{4}]\},$$

7 $${t}_{1}=2p\alpha \beta {|{\xi }_{0}(t)|}^{2},$$

8 $${t}_{2}=-2p\alpha \beta {|{\xi }_{0}(t)|}^{2},$$

9 $${t}_{3}=p\{{\alpha }^{2}+{\beta }^{2}{[{|{\xi }_{1}(t)|}^{2}-{|{\xi }_{0}(t)|}^{2}]}^{2}\}.$$

Similarly, the five characterizing parameters of the reduced density matrix ${\rho }_{ab}(t)$ for the reservoir-subsystem *ab* in the Bloch decomposition are

10 $$x=p\{{\alpha }^{2}+{\beta }^{2}[{|{\xi }_{0}(t)|}^{4}-{|{\xi }_{1}(t)|}^{4}]\},$$

11 $$y=p\{{\alpha }^{2}+{\beta }^{2}[{|{\xi }_{0}(t)|}^{4}-{|{\xi }_{1}(t)|}^{4}]\},$$

12 $${t}_{1}=2p\alpha \beta {|{\xi }_{1}(t)|}^{2},$$

13 $${t}_{2}=-2p\alpha \beta {|{\xi }_{1}(t)|}^{2},$$

14 $${t}_{3}=p\{{\alpha }^{2}+{\beta }^{2}{[{|{\xi }_{0}(t)|}^{2}-{|{\xi }_{1}(t)|}^{2}]}^{2}\}.$$

Based on the definition in the Methods section, we can readily obtain the geometric discords of the qubit-subsystem and the reservoir-subsystem.

**Figure 1:**
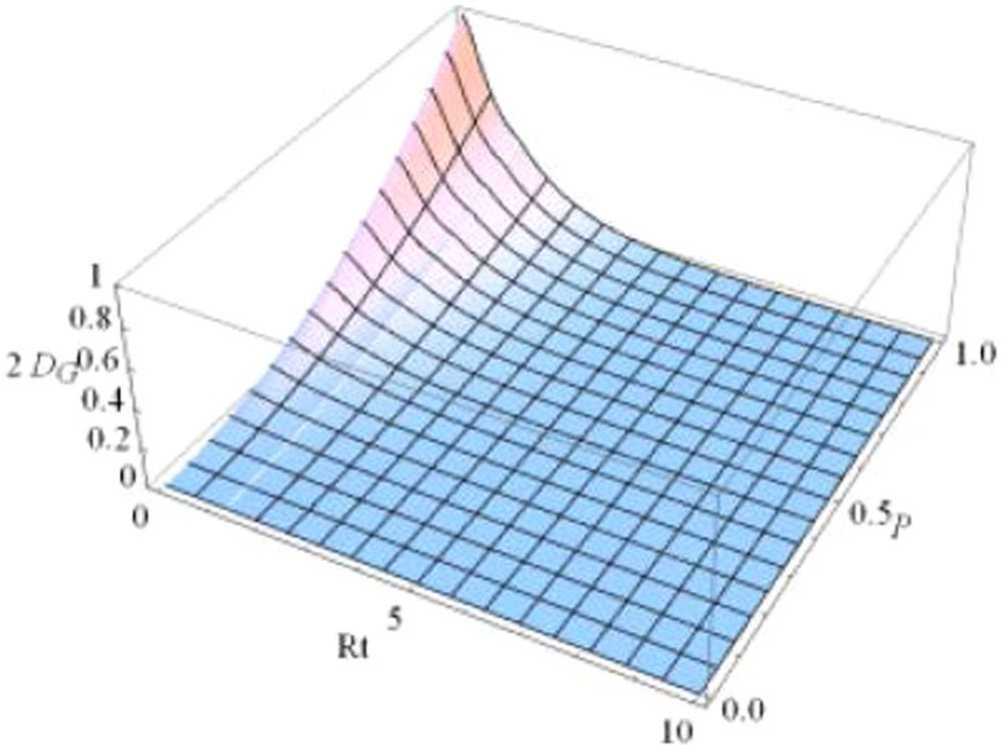
Geometric discord of qubit-subsystem *AB* as a function of the dimensionless time *Rt* and the parameter *p* under the Markovian $({\rm{\Gamma }}=5R)$ regime with $\alpha =1/\sqrt{2}$.

**Figure 2:**
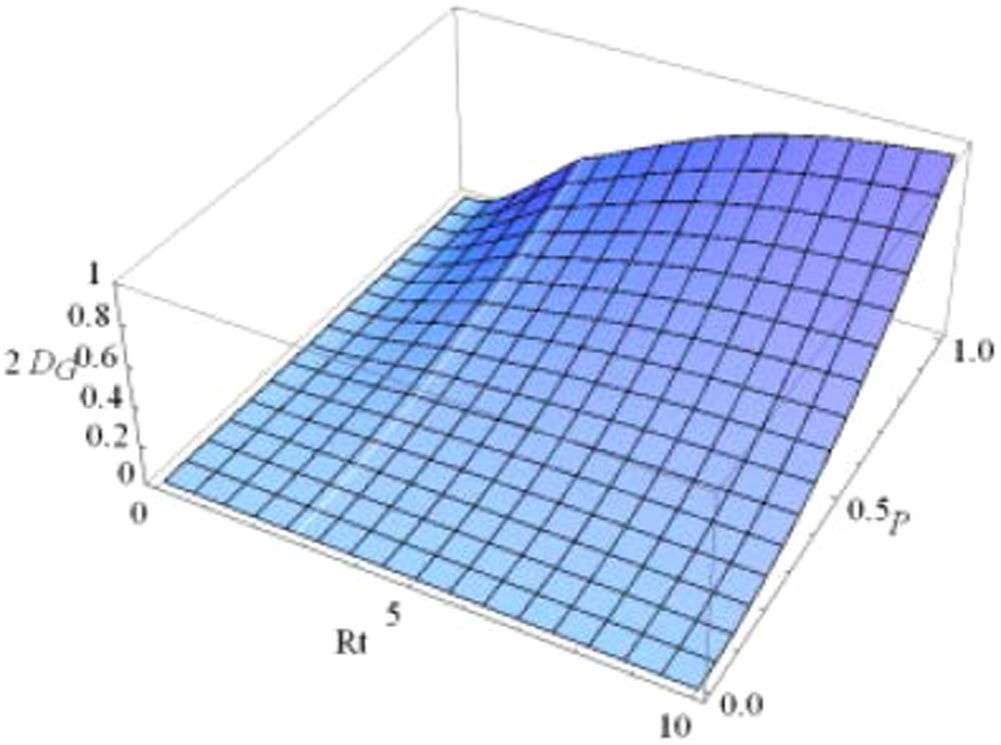
Geometric discord of reservoir-subsystem *ab* as a function of the dimensionless time *Rt* and the parameter *p* under the Markovian $({\rm{\Gamma }}=5R)$ regime with $\alpha =1/\sqrt{2}$.

**Figure 3:**
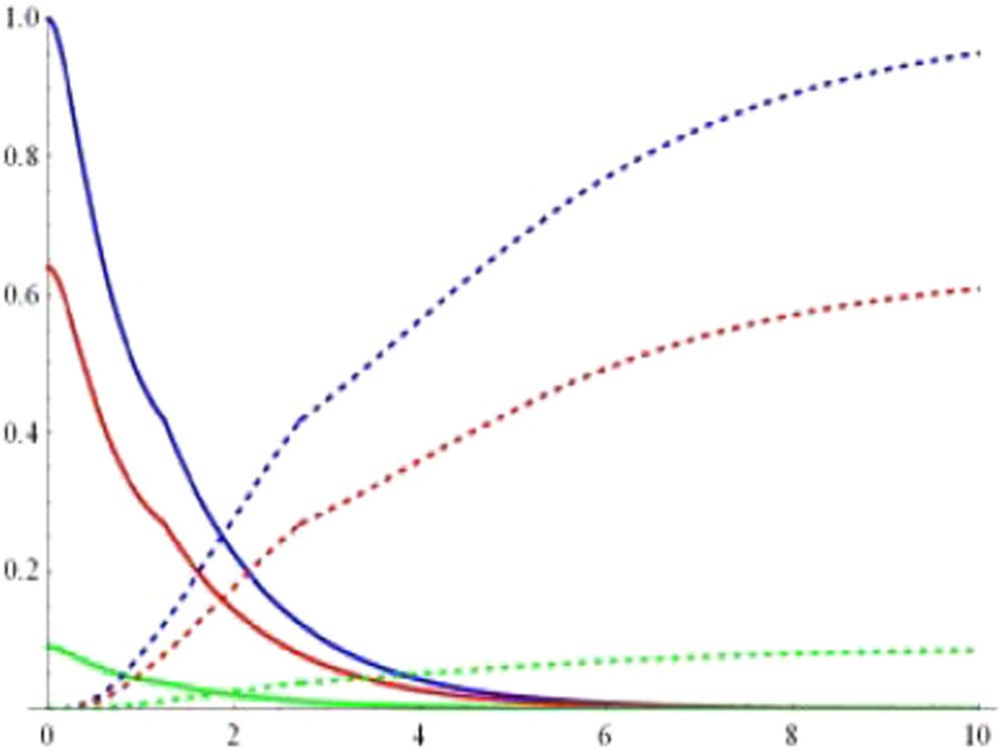
Geometric discord of the qubit-subsystem *AB* (solid line) and the corresponding reservoir-subsystem ab (dashed line) as a function of the dimensionless time *Rt* for the parameter *p* = 1 (blue line), *p* = 0.8 (red line) and *p* = 0.3 (green line) under the Markovian $({\rm{\Gamma }}=5R)$ regime with $\alpha =1/\sqrt{2}$.

To display the dynamical behaviors of geometric discord, we first plot the geometric discords as a function of the dimensionless time *Rt* and the parameter *p* under the Markovian regime $({\rm{\Gamma }}=5R)$ with $\alpha =1/\sqrt{2}$ in Figs 1 and 2. Obviously, one can find that the geometric discord of qubit-subsystem ${\rho }_{AB}(t)$ as shown in Fig. 1 decays asymptotically to zero, while that of reservoir-subsystem ${\rho }_{ab}(t)$ as shown in Fig. 2 arises from time *t* = 0. To clearly show the relationships, we plot the geometric discord of the qubit-subsystem ${\rho }_{AB}(t)$ (solid line) and the corresponding reservoir-subsystem ${\rho }_{ab}(t)$ (dashed line) as a function of the dimensionless time *Rt* for different initial purity in terms of *p* under the Markovian regime in Fig. 3. It directly shows that there exhibits a clear transfer of geometric discord from the qubit- to the reservoir-subsystem. Furthermore, the amounts of both geometric discords depend on the purity *p*. For example, in the case of $\alpha =1/\sqrt{2}$, the smaller the value of *p* can induce the smaller amount of geometric discord. In particular, it is interesting to note that the discords undergo sudden changes in the time evolution independent on the purity *p*. Actually, the so-called “sudden change of geometric discord” is just a signature of the jump of the time derivative of that function in a specific instant. We know that the geometric discord can be explained as a minimal distance from the set of classical-quantum state. When the distance to the classical-quantum state remains constant the distance function is constant, while there will come out a sudden change at the transition point when the distance function is optimized by the time-dependent classical-quantum state in a certain dynamical evolution. This reveals that the location of classical-quantum state changes in a discontinuous way in the vicinity of transition point. Actually, the derivation of such features came out as a consequence of the negligibility of the set of zero discord states in comparison with the whole state space, which renders the robustness of geometric discord decoherent.

**Figure 4:**
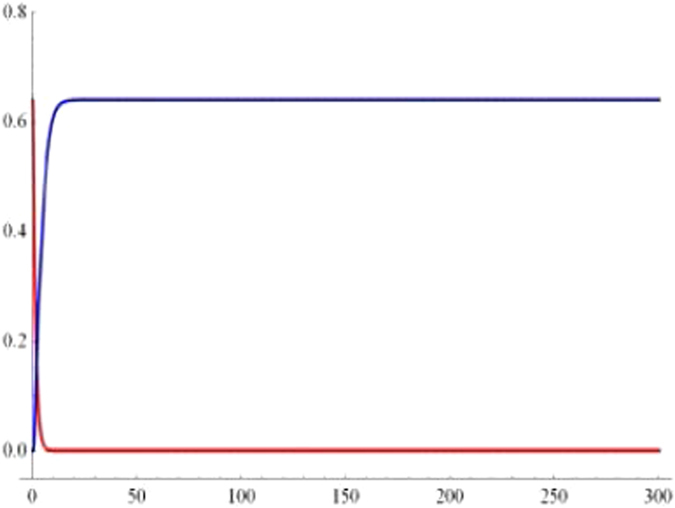
The long-term behaviour of geometric discord for qubit-subsystem AB (red line) and the corresponding reservoir-subsystem ab (blue line) as a function of the dimensionless time *Rt* under the Markovian $({\rm{\Gamma }}=5R)$ regime with $p=0.8$ and $\alpha =1/\sqrt{2}$.

In order to examine the long-term behaviors of geometric discord, we plot the geometric discord of the qubit-subsystem *AB* (red line) and that of reservoir-subsystem *ab* (blue line) as a function of the dimensionless time *Rt* under the Markovian regime with *p* = 0.8 and $\alpha =1/\sqrt{2}$ in Fig. 4. One can see that the geometric discord of qubit-subsystem decays asymptotically to zero exhibiting the Markovian effects of the reservoir. On account of the information transfer, the geometric discord of reservoir-subsystem revives from zero to a fixed value.

### The dynamics of geometric discord and its transfer for W-class state

Next, let us proceed by discussing on the dynamics of the geometric discord for the extended W-class state. We take into account a tripartite qubit system composed by three noninteracting qubits *A*, *B* and *C* each individually interacting with an independent multimode reservoir labeled as *a*, *b* and *c*. Assuming the reservoir-subsystem is initially prepared in the vacuum states ${|\bar{0}\bar{0}\bar{0}\rangle }_{abc}$ and the qubit-subsystem is initially in the extended W-class state^42^

15 $${\rho }_{ABC}(0)=\frac{1-p}{8}I+p|W(0)\rangle \langle W(0)|,$$

where *I* is an 8 × 8 identity matrix, the complex numbers *α*, *β* and *γ* are the state parameter contained in the W-like state $|W(0)\rangle =\alpha |100\rangle +\beta |010\rangle +\gamma |001\rangle$ with ${|\alpha |}^{2}+{|\beta |}^{2}+{|\gamma |}^{2}=1$, the real number parameter *p* also indicates the purity of the initial state (*p* is 1 for pure sate and 0 for completely mixed state). Particularly, an interesting property of the *W*-class state is that it remains the bipartite entanglement when anyone of the three qubits is traced out. That is, the *W*-class state contains only pairwise entanglement between any two qubits, it is reasonable to consider the dynamics of bipartite geometric discord contained in this state.

The reduced matrix operator of qubit-subsystem *AB* can be obtained by tracing the total evolved state ${\rho }_{ABCabc}(t)$ over the qubit *C* and the reservoirs *abc*. Making use of the methods mentioned above, the five characterizing parameters of the reduced density matrix ${\rho }_{AB}(t)$ for the qubit-subsystem in Bloch decomposition are

16 $$x=p[({\alpha }^{2}-{\beta }^{2}+{\gamma }^{2}){|{\xi }_{0}(t)|}^{2}+{|{\xi }_{1}(t)|}^{2}],$$

17 $$y=p[({\alpha }^{2}+{\beta }^{2}-{\gamma }^{2}){|{\xi }_{0}(t)|}^{2}+{|{\xi }_{1}(t)|}^{2}],$$

18 $${t}_{1}=2p\beta \gamma {|{\xi }_{0}(t)|}^{2},$$

19 $${t}_{2}=2p\beta \gamma {|{\xi }_{0}(t)|}^{2},$$

20 $${t}_{3}=p[({\alpha }^{2}-{\beta }^{2}-{\gamma }^{2}){|{\xi }_{0}(t)|}^{2}+{|{\xi }_{1}(t)|}^{2}].$$

Similarly, the five characterizing parameters of the reduced density matrix ${\rho }_{ab}(t)$ in Bloch decomposition for the reservoir-subsystem are

21 $$x=p[({\alpha }^{2}-{\beta }^{2}+{\gamma }^{2}){|{\xi }_{1}(t)|}^{2}+{|{\xi }_{0}(t)|}^{2}],$$

22 $$y=p[({\alpha }^{2}+{\beta }^{2}-{\gamma }^{2}){|{\xi }_{1}(t)|}^{2}+{|{\xi }_{0}(t)|}^{2}],$$

23 $${t}_{1}=2p\beta \gamma {|{\xi }_{1}(t)|}^{2},$$

24 $${t}_{2}=2p\beta \gamma {|{\xi }_{1}(t)|}^{2},$$

25 $${t}_{3}=p[({\alpha }^{2}-{\beta }^{2}-{\gamma }^{2}){|{\xi }_{1}(t)|}^{2}+{|{\xi }_{0}(t)|}^{2}].$$

**Figure 5:**
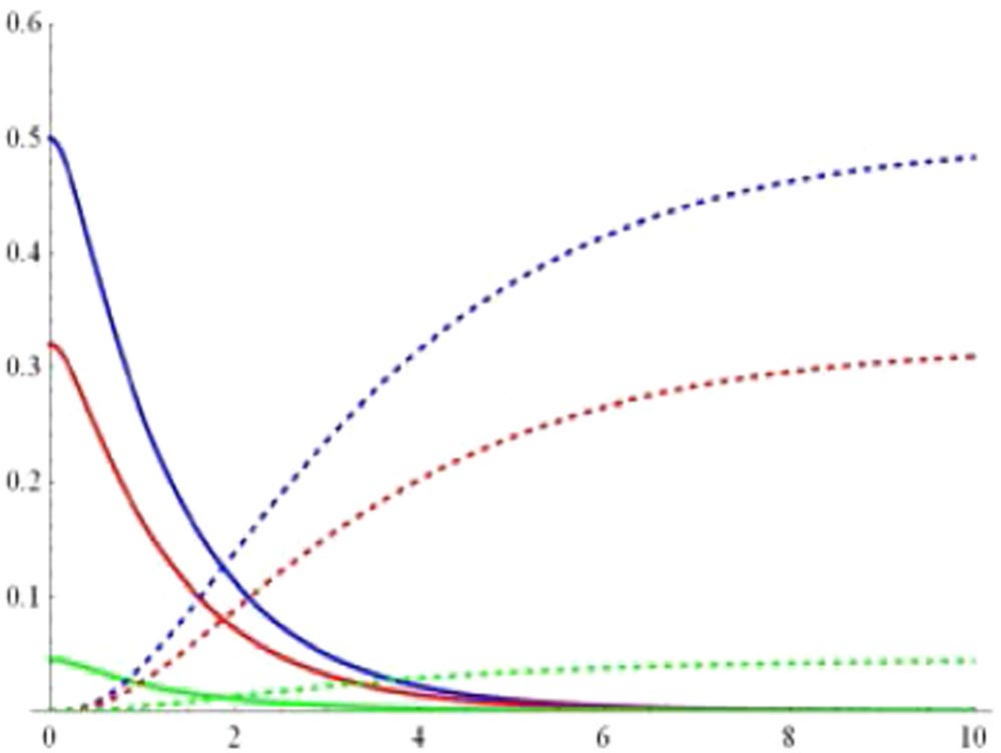
Geometric discord of the qubit-subsystem AB (solid line) and the corresponding reservoir-subsystem ab (dashed line) as a function of the dimensionless time *Rt* for the parameter *p* = 1 (blue line), *p* = 0.8 (red line) and *p* = 0.3 (green line) under the Markovian $({\rm{\Gamma }}=5R)$ regime with $\alpha =\beta =1/2$, $\gamma =\sqrt{2}/2$.

**Figure 6:**
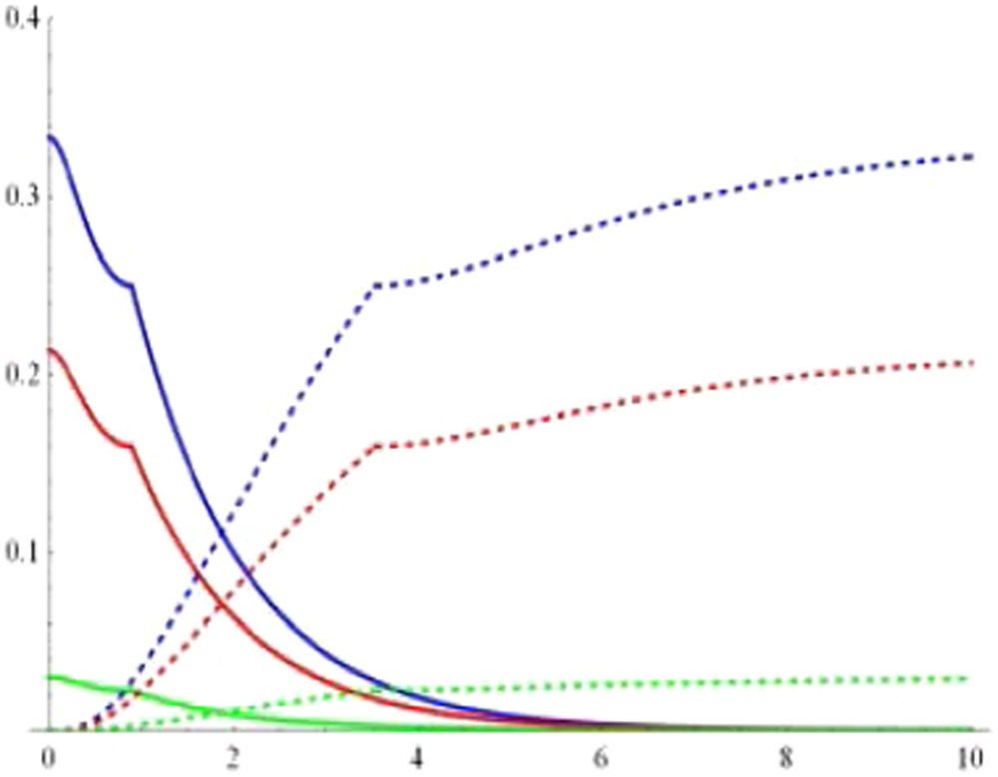
Geometric discord of the qubit-subsystem AB (solid line) and the corresponding reservoir-subsystem ab (dashed line) as a function of the dimensionless time *Rt* for the parameter *p* = 1 (blue line), *p* = 0.8 (red line) and *p* = 0.3 (green line) under the Markovian $({\rm{\Gamma }}=5R)$ regime with $\alpha =\beta =\gamma =1/\sqrt{3}$.

**Figure 7:**
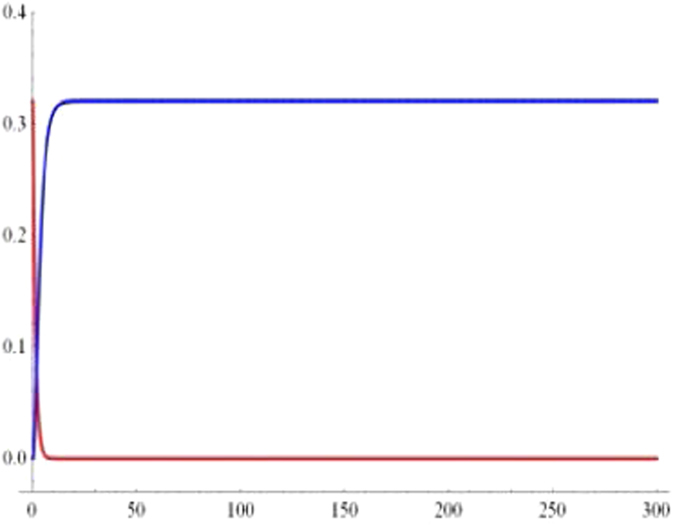
The long-term behaviour of geometric discord for qubit-subsystem AB (red line) and the corresponding reservoir-subsystem ab (blue line) as a function of the dimensionless time *Rt* under the Markovian $({\rm{\Gamma }}=5R)$ regime with *p* = 0.8 and $\alpha =\beta =1/2$, $\gamma =\sqrt{2}/2$.

Upon the above preparations, one can readily obtain the geometric discords of the qubit- and that of the reservoir-subsystem. As plotted in Figs 5 and 6, we depict their dynamical behaviors of the qubit-subsystem *AB* (solid line) and the corresponding reservoir-subsystem *ab* (dashed line) as a function of the dimensionless time *Rt* for the different parameter *p* within the Markovian regime when the state parameters are chosen as $\alpha =\beta =1/2$, $\gamma =\sqrt{2}/2$ and $\alpha =\beta =\gamma =1/\sqrt{3}$, respectively. Clearly, one can see that the geometric discord of qubit-subsystem decays asymptotically to zero and that of the corresponding reservoir-subsystem revives from time *t* = 0, which straightforward shows a transfer of discord from the qubit- to the reservoir-subsystem. The amount of both geometric discords depend on the purity *p*. For example, in the case of $\alpha =\beta =1/2$, $\gamma =\sqrt{2}/2$ as shown in Fig. 5, the smaller the value of *p* can result in the smaller the amount of geometric discords. Moreover, the amount of geometric discords of the qubit- and the reservoir-subsystem also depends on the values of *α, β*, *γ*, i.e., when *p* = 1 (see Figs 5 and 6), the amount of geometric discords for $\alpha =\beta =1/2$, $\gamma =\sqrt{2}/2$ is larger than that of it for $\alpha =\beta =\gamma =1/\sqrt{3}$. With this in mind, we can conclude that the *W*-class state taking the form of ${|W\rangle }_{ABC}=\frac{1}{2}(|100\rangle +|010\rangle +\sqrt{2}|001\rangle )$ is more robust and useful than the form of ${|W\rangle }_{ABC}=\frac{1}{\sqrt{3}}(|100\rangle +$ $|010\rangle +|001\rangle )$ during quantum information-processing tasks. What’s more, the discords of qubit- and the reservoir-subsystem exist sudden changes during the time evolution in the case of $\alpha =\beta =\gamma =1/\sqrt{3}$ (in Fig. 6), contrarily such phenomenon cannot take place when $\alpha =\beta =1/2$, $\gamma =\sqrt{2}/2$ (in Fig. 5). Finally, we also exploit the long-term behaviors of the geometricdiscords. In Fig. 7, we plot the geometric discords of qubit-subsystem (red line) and the corresponding reservoir-subsystem (blue line) as a function of the dimensionless time *Rt* under Markovian regime with *p* = 0.8 and $\alpha =1/\sqrt{2}$. It is apparent that the geometric discord in qubit-subsystem decays asymptotically to zero, exhibiting the Markovian effect of reservoir, while one of reservoir-subsystem revives from zero to fixed value due to the information transfer.

## Discussions

In conclusion, we have investigated the dynamics of geometric discord and its transfer under the dissipative reservoirs. Firstly, we consider the first model consisting of two noninteracting qubits *A*, *B* being initially in an extended Werner-like state^35^ and each locally coupled with its own multimode reservoirs. The results show that the geometric discord of the qubit-subsystem decays asymptotically to zero and one of the reservoir-subsystem revives from time *t* = 0, which exhibits a clear transfer of discord from the qubit- to the reservoir-subsystem. Moreover, both geometric discords undergo sudden changes during the evolution, and their amounts depend on the purity *p*. Further, we consider the other model, i.e., three qubits *A*, *B* and *C* being initially in a tripartite correlated state and interplaying with independent reservoirs, respectively. It has been shown that the geometric discord of qubit-subsystem decays asymptotically to zero and one of the corresponding reservoir-subsystem will revives from zero to steady value, exhibiting a clear transfer of geometric discord from qubit- to reservoir-subsystem. Moreover, both the amount of geometric discords depend on both the purity *p* and the state parameters *α*, *β*, *γ* as well. Interestingly, the discords undergo sudden changes during the evolution in the case of $\alpha =\beta =\gamma =1/\sqrt{3}$ (as shown in Fig. 6), while this never occurs in the case of $\alpha =\beta =1/2$, $\gamma =\sqrt{2}/2$ (as shown in Fig. 5). We also investigate the long-term behaviors of geometric discords contained in both models, and it shows that the geometric discord of qubit-subsystem decays asymptotically to zero and that of the reservoir-subsystem revives from zero to steady value. As mentioned above, QC is expected to play a key role for various practical quantum tasks, thus, it is fundamentally important to explore its dynamics in terms of the geometric discord, and we claim that our results would be helpful for an insight into the dynamics of quantum correlation during quantum information processing and understanding some related concepts of quantum mechanics.

## Methods

### Briefly review of the geometric discord and the physical model

In this section, our aim is to introduce the descriptions of the geometric discord and the physical model of the quantum-reservoir system. To begin with, we provide a brief review for the geometric discord based on the Hilbert-Schmidt norm by Dakić *et al*.^12, 27^

26 $${D}^{G}(\rho )\,:=\mathop{{\rm{\min }}}\limits_{\chi \in {\rm{\Theta }}}{\Vert \rho -\chi \Vert }^{2},$$

where ${\Vert \rho -\chi \Vert }^{2}=Tr{(\rho -\chi )}^{2}$ is the square of Hilbert-Schmidt norm of Hermitian operators and Θ denotes the set of zero-discord states. In general, a given state is classically correlated when it can be written as

27 $${\rho }_{cc}=\sum _{i,j}{p}_{ij}|{i}^{A}\rangle \langle {i}^{A}|\otimes |{j}^{B}\rangle \langle \,{j}^{B}|,$$

where $\{|{i}^{A}\rangle ,|{j}^{B}\rangle \}$ are sets of orthogonal states of party *A* and *B*, with non-negative probabilities *p*_*ij*_ satisfying ${\sum }_{i,j}{p}_{ij}=1$. That is to say, a quantum state has zero discord with respect to all bipartitions if and only if it can be written in the classical-quantum form as shown in Eq. (27). A necessary and sufficient condition for nonzero quantum discord was proposed in ref. 27, which can be easily extended to the concept of QCs. For any two-qubit state, a closed form of expression for the geometric discord can be obtained as

28 $${D}^{G}(\rho )=\frac{1}{4}({\Vert \mathop{x}\limits^{\rightharpoonup }\Vert }^{2}+{\Vert T\Vert }^{2}-{\lambda }_{{\rm{\max }}}),$$

where ${x}_{i}=Tr({\sigma }_{i}^{A}\rho )$ are components of the local Bloch vector for subsystem *A*, ${T}_{ij}=Tr\rho ({\sigma }_{i}\otimes {\sigma }_{j})$ are components of the correlation tensor, and ${\sigma }_{i}(i=1,2,3)$ are the three standard Pauli matrices. The vector $\mathop{x}\limits^{\rightharpoonup }:={({x}_{1},{x}_{2},{x}_{3})}^{t}$ is a column vector, parameter $T:=({T}_{ij})$ denotes a matrix, and ${\lambda }_{\max }$ is the largest eigenvalue of the matrix $K=\mathop{x}\limits^{\rightharpoonup }{\mathop{x}\limits^{\rightharpoonup }}^{t}+T{T}^{t}$ with the superscript *t* denoting transpose. It is worth noting that geometric discord *D* ^*G*^ is not normalized to 1: for two-qubit state, its maximum value is $1/2$.

For a general two-qubit *X* state, the density matrix in the representation spanned by $\{|00\rangle \equiv |1\rangle ,|01\rangle \equiv |2\rangle ,|10\rangle \equiv |3\rangle ,|11\rangle \equiv |4\rangle \}$ is given by

29 $$\rho =(\begin{array}{cccc}{\rho }_{11} & 0 & 0 & {\rho }_{14}\\ 0 & {\rho }_{22} & {\rho }_{23} & 0\\ 0 & {\rho }_{23}^{\ast } & {\rho }_{33} & 0\\ {\rho }_{14}^{\ast } & 0 & 0 & {\rho }_{44}\end{array}),$$

where there are seven real parameters. Up to local unitary equivalence, we can assume that ${\rho }_{14}$ and ${\rho }_{23}$ are also real, and in fact there are only five independent parameters (note that quantum discord is invariant under local unitary transformations). Alternatively, if we represent the *X*-state in the Bloch decomposition, then the five characterizing parameters can be expressed as refs 45, 46

30 $$x=Tr({\sigma }_{z}^{A}\rho )={\rho }_{11}+{\rho }_{22}-{\rho }_{33}-{\rho }_{44},$$

31 $$y=Tr({\sigma }_{z}^{B}\rho )={\rho }_{11}-{\rho }_{22}+{\rho }_{33}-{\rho }_{44},$$

32 $${t}_{1}=Tr({\sigma }_{x}^{A}{\sigma }_{x}^{B}\rho )=2({\rho }_{14}+{\rho }_{23}),$$

33 $${t}_{2}=Tr({\sigma }_{y}^{A}{\sigma }_{y}^{B}\rho )=2(-{\rho }_{14}+{\rho }_{23}),$$

34 $${t}_{3}=Tr({\sigma }_{z}^{A}{\sigma }_{z}^{B}\rho )={\rho }_{11}-{\rho }_{22}-{\rho }_{33}+{\rho }_{44}.$$

As shown in Eq. (29) $\rho$ is a two-qubit *X* state and its characterizing parameters are defined in Eqs (30–34), we have $\mathop{x}\limits^{\rightharpoonup }={(0,0,x)}^{t}$ and $T=diag\{{t}_{1},{t}_{2},{t}_{3}\}$, and then the geometric discord of the two-qubit *X* state can be obtained by

35 $${D}^{G}(\rho )=\frac{1}{4}({t}_{1}^{2}+{t}_{2}^{2}+{t}_{3}^{2}+{x}^{2}-\,{\rm{\max }}\{{t}_{1}^{2},{t}_{2}^{2},{t}_{3}^{2}+{x}^{2}\}).$$

Now, we describe the physical model where the interaction between a single qubit and multimode reservoir can be depicted by the spin-boson model. The Hamiltonian of individual qubit–reservoir subsystem can be expressed as the spin-boson model ($\hslash =1$)^43, 44^

36 $${H}_{Xx}={\omega }_{0}{\sigma }_{+}^{X}{\sigma }_{-}^{X}+\sum _{j}{\omega }_{j}{x}_{j}^{\dagger }{x}_{j}+\sum _{j}({g}_{j}{\sigma }_{+}^{X}{x}_{j}+{g}_{j}^{\ast }{\sigma }_{-}^{X}{x}_{j}^{\dagger }),$$

where ${g}_{j}$ is coupling constant for the qubit and the reservoir *j*-th mode, ${x}^{\dagger }$(*x*) are the creation (annihilation) operators of the reservoir with the frequency ${\omega }_{j}$, ${\sigma }_{+}^{X}=|1\rangle \langle 0|$ and ${\sigma }_{-}^{X}=|0\rangle \langle 1|$ are the rising and falling operators of the qubit acting on the *X*th qubit with transition frequency ${\omega }_{0}$, in which $|0\rangle$ and $|1\rangle$ denoting the orthogonal computational basis. The evolution of a single qubit and multimode reservoir under the system-environment coupling depends on the particular choice of the Lorentzian spectral density of the reservoir. In this case, based on the non-perfect reflectivity of the cavity mirrors the fundamental mode ${\omega }_{c}$ supported by the cavity displays a Lorentzian broadening, the Lorentzian spectral distribution of the following form refs 43, 44

37 $$J(\omega )=\frac{{R}^{2}}{\pi }\frac{{\rm{\Gamma }}}{{(\omega -{\omega }_{c})}^{2}+{{\rm{\Gamma }}}^{2}}.$$

In equation (37), the parameter R can be shown to be connected to the qubit-cavity coupling strength and ${\rm{\Gamma }}$ denotes the half-width at half-maximum of the intracavity field spectrum profile. Moreover, the relation between the parameters and the cavity correlation function is $f(t-t^{\prime} )={R}^{2}\exp [-{\rm{\Gamma }}(t-t^{\prime} )]$. For the above scenario of a single qubit and multimode reservoir coupling, the evaluations of the different initial states have already been discussed in the literatures^43, 44^, which are

38 $${|0\rangle }_{X}{|\overline{0}\rangle }_{x}\to {|0\rangle }_{X}{|\overline{0}\rangle }_{x},$$

39 $${|1\rangle }_{X}{|\overline{0}\rangle }_{x}\to {\xi }_{0}(t){|1\rangle }_{X}{|\overline{0}\rangle }_{x}+{\xi }_{1}(t){|0\rangle }_{X}{|\overline{1}\rangle }_{x},$$

where ${\xi }_{0}(t)={e}^{-{\rm{\Gamma }}t/2}[\cosh (\frac{dt}{2})+\frac{{\rm{\Gamma }}}{d}\,\sinh (\frac{dt}{2})]$ and ${|{\xi }_{0}(t)|}^{2}+{|{\xi }_{1}(t)|}^{2}=1$ with $d=\sqrt{{{\rm{\Gamma }}}^{2}-4{R}^{2}}$. Herein, we emphasize that for the independent qubit–reservoir system the analytical solutions of $\rho (t)$ can be derived for arbitrary initial states. Therefore, in this paper, we can calculate the geometric discord of the arbitrary bipartite subsystem by means of the exact solutions.

## Electronic supplementary material

Supplementary Dataset 1
