# Supplementary material for: Quantum correlation of qubit-reservoir system in dissipative environments
Source: Sci Rep. 2017 Aug 17;7:8625. doi: 10.1038/s41598-017-07235-3 (PMC5561145; doi:10.1038/s41598-017-07235-3)
Supplement: Supplementary file 1 — Supplementary Dataset 1 [file 41598_2017_7235_MOESM1_ESM.doc]

**Quantum correlation of qubit-reservoir system in dissipative environments**

Tao Wu1, 2[[1]](#footnote-2)*, Jiadong Shi^1, 2, 3^, Lizhi Yu^1, 2^, Juan He^1, 2^ & Liu Ye^3^

^1^School of Physics & Electronics Engineering, Fuyang Normal University, Fuyang,

236037, China

^2^Research Centre of Quantum Information Technology, Fuyang Normal University, Fuyang,

236037, China

^3^School of Physics & Material Science, Anhui University, Hefei. Anhui 230601, China

**Appendix**

**Revisiting the evolution of global qubit-reservoir state.** In this Appendix, we aim to revisit the evolution of the global qubit-reservoir state, and will take the EWL state as an example. For the case that the qubits $A$ and $B$ are initially prepared in the EWL state, and the reservoirs $a$ and $b$ are initially in the vacuum states, the initial density matrix of the total qubit-reservoir system thus reads as

$\rho_{ABab}\left(0\right)=\rho_{AB}\left(0\right)\otimes|\bar{0}\bar{0}\rangle_{ab}\langle\bar{0}\bar{0}|,$ (1)

which will evolve to

(2)

with

. (3)

By tracing over the total evolved state $\rho_{ABab}\left(t\right)$ over the reservoir-subsystem, one can obtain the reduced matrix operators of the qubit-subsystem $\rho_{AB}\left(t\right)$, which is the same for the obtained reduced matrix operator of the reservoir-subsystem $\rho_{ab}\left(t\right)$. In addition, for the extended W-class state, the evolution of the global qubit-reservoir state can also be obtained by using the processes as given by Eqs. (1-3).

1. ^*^**Corresponding author**: wutaofuyang@126.com [↑](#footnote-ref-2)
